# Evaluation of Methods for Collecting Mosquitoes (Culicidae: Diptera) in Canopy and Ground Strata in the Brazilian Savanna

**DOI:** 10.3390/tropicalmed7120446

**Published:** 2022-12-19

**Authors:** Luis Filipe Mucci, Eduardo Sterllino Bergo, Juliana Telles de Deus, Simone Luchetta Reginato, Mariza Pereira, Vera Lucia Fonseca de Camargo-Neves

**Affiliations:** Secretaria de Estado da Saúde de São Paulo, Instituto Pasteur, São Paulo 01027-000, SP, Brazil

**Keywords:** diurnal mosquitoes, kairomones, canopy stratum, Brazilian savanna

## Abstract

The hand-net is the standard method for capturing mosquitoes with sylvatic diurnal activity in disease outbreaks in Brazil. However, occupational risks and biases related to the collectors’ abilities and attractiveness are important limitations. In this study, we compared hand-nets with automatic traps (CDC) associated to CO_2_ and BG-Lure^®^ in the Vassununga State Park, a Brazilian Savanna protection area. The collections carried out over 27 days on the ground and the forest canopy. A total of 1555 mosquitoes were obtained in 20 taxa. The diversity index ranged between 1.12 and 1.79 and the dominance index from 0.22 to 0.40. The dominant species on the ground was *Aedes scapularis* (46.0%), and in the canopy, *Hg. janthinomys/capricornii* (31.9%). *Haemagogus leucocelaenus* was rare (*n* = 2). The hand-net resulted in the greatest diversity and abundance of species in both strata, followed by the traps associated with CO_2_. A low degree of similarity was observed between the hand-net on the ground compared to the other capture methods. The use of BG-Lure^®^ alone resulted in a low number of specimens. In conclusion, the hand-net is still the method of choice for collecting arbovirus vectors in the diurnal period, especially yellow fever vectors.

## 1. Introduction

In Brazil, many species of Culicidae (Diptera) are of medical interest [1,2] and much of the knowledge about their bioecology and epidemiological role is the result of research and entomological surveys associated with outbreaks of arboviruses [3,4,5,6,7]. In these studies, the mobile human attraction technique using capture hand-nets was widely used, often in entomological captures in the forest canopy, aimed at increasing the sample of mosquitoes that feed on the blood of birds and nonhuman primates, which are considered important hosts for several arboviruses [5,8,9]. For this reason, in Brazil, the hand-net was adopted as the standard technique by health surveillance services across the country, with canopy capture recommended as a complementary procedure [10].

However, in captures with humans beings, the attractants are subject to variations in individual performance and the attractiveness of each collector, which can imply a bias resulting in sample divergences [11]. Moreover, collections performed by individuals raises concerns about occupational risks [12,13], especially when working at heights in tree canopies, according to the specific safety protocols of each country.

Thus, there is a need for alternative techniques to replace the standard hand-net technique that can ensure representativeness in mosquito sampling. Service (1993) and Santos et al. (2021) point out that the selection of the appropriate method should take into account the specific characteristics of the natural history, biology, and ecology of the target species of potential vectors [14,15].

One of the alternative techniques is the use of automatic suction traps with a luminous attractant, commonly used to collect nocturnal species [13,16]. In Brazil, the association of a carbon dioxide source and other types of odor attractants (such as octenol and lactic acid) have been used to increase the sensitivity of this type of trap for daytime use [17,18,19,20,21]. Other studies were restricted to afternoon and nocturnal periods [22,23,24,25,26], or focused on the urban environment, for capturing *Aedes aegypti* and *Culex quinquefasciatus* [27,28,29].

Recently, our group began looking for alternative and/or complementary methods for daytime collections in accordance with forest stratification to carry out routine surveys in different biomes. In a previous study, we conducted a comparative evaluation of in-person capture using hand-nets and automatic traps with carbon dioxide (dry ice) and BG-Lure^®^ as attractants on the ground and in the canopy stratum of an Atlantic Forest environmental reserve [21]. In the present study, we apply the same methodology used in the previous study for a comparative evaluation of these methods in the Cerrado biome.

## 2. Materials and Methods

### 2.1. Research Area

**Figure 1:**
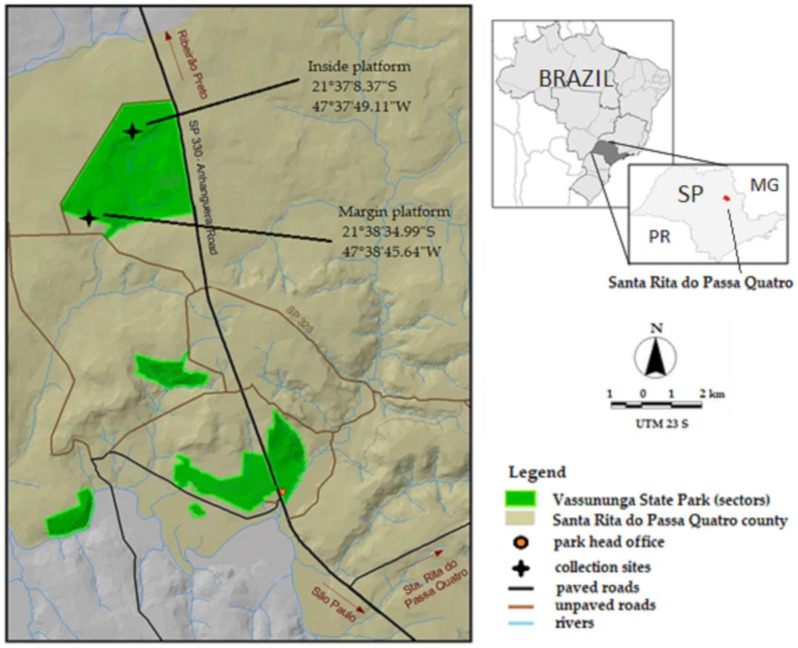
Location of the study area in the savanna biome, Vassununga State Park. Source: Plano de Manejo PEV, 2009.

The Vassununga state park (Parque Estadual Vassununga—PEV) is a protected conservation area that was created to protect representative areas of the seasonal semideciduous forest (inland Atlantic Forest) and different “Cerrado” (Brazilian savanna) physiognomies. This park, made up of six discontinuous sectors with a total area of 2071.42 hectares, is located in the municipality of Santa Rita do Passa Quatro, São Paulo, Brazil [30] (Figure 1). The Pé-do-Gigante sector, chosen for this study, is the largest area in the Park (1212.92 ha) and contains the three types of savanna: forested savanna (“Cerradão”), arborized savanna (“Cerrado *stricto sensu*”), and savanna “Gramíneo-Lenhosa” (grassy-shrub), according to the classification adopted by Veloso et al. (1991) [31,32].

According to the Köppen climate classification, the climate of the Santa Rita do Passa Quatro region is Cwa: rainy in summer and dry in winter. The average annual temperature is 23.3 °C, with an average maximum temperature of approximately 26.0 °C in summer (December to February) and an average minimum temperature of approximately 19.0 °C in winter (June to August). According to the Park Management Plan [30], the park has an average annual rainfall of 1365.7 mm and potential evapotranspiration of 1160.61 mm.

Two sites in the area were selected for the collections: the forest margin and inside the forest (about 3 km apart). In each site, collections were carried out in the forest canopy and at ground level. For the sampling of mosquitoes at the edge of the forest, a platform was installed at the first branch ramification of the tree canopy, seven meters above the ground, accessed using ropes and climbing equipment by a trained capturer (File S1). This platform is in an area characterized as arborized savanna (“Cerrado *ss*”), a dense savanna with a predominantly arboreal vegetation subtype, with 50 to 70% coverage and an average height of five to eight meters. This physiognomy represents the highest and densest form of savanna. In the second sampling area, inside the forest, we used the platform of a meteorological tower. The capturer worked at a height of eight meters, corresponding to the average canopy height of the surrounding trees. This sector of the park corresponds to an area of dense savanna transitioning into “Cerradão”, with a predominance of trees in a closed canopy that approaches forest vegetation, with an average tree height of 10 to 12 m [30].

### 2.2. Capture of Specimens

Entomological captures were carried out on three consecutive days per month in February, March, October, November, and December of 2020, and in January, February, April, and May of 2021. The pause in collections was due to COVID-19 pandemic restrictions. Two techniques were used:

#### 2.2.1. Capture by Hand-Net

Mosquitoes were captured with hand-nets and sucked with a mouth aspirator, where they were retained until the end of the collection period (morning or afternoon) [10]. A detailed description of the equipment can be seen in the File S2. Captures were carried out from 9:00 to 12:00 am and 1:00 to 4:00 pm in both sectors simultaneously. In each sector, the collections were carried out by two collectors, one on the platform and another at ground level. On the ground, the collector covered approximately 1500 m of trails around each platform. The collectors rotated the stratum between 12:00 am and 1:00 pm when they also stored the specimens collected in the morning. At the end of the afternoon period, at 4:00 pm, the same procedure to store the samples was performed, which was important to avoid damage to the morphological structures and to be able to freeze the insects still alive. The insects collected were transferred to cryotubes and frozen alive in liquid nitrogen to preserve viral genetic material for future analysis.

#### 2.2.2. Capture with Automatic Traps

CDC-type electric traps [33] were used with lights off and two chemical attractants: carbon dioxide (dry ice) and BG-Lure^®^ (a commercial product), which is a chemical attractant composed of ammonia, L- lactic acid, and caproic acid. In each sector (forest margin and interior), four traps were installed close to the ground, between 1 and 1.5 min height, and another four in the tree canopy at a minimum height of 5 m, depending on the average height of the trees at the site. There were sixteen traps in total, with eight positioned at the forest edge and eight inside the forest around each platform, considered the central reference point (Figure 1). The traps were placed at points to the north, south, east, and west, at approximately 250 m from the platform, and each day the traps were rotated to alternate the position of the exposure of the attractants. One pair of traps (ground–canopy) was used with only CO_2_, another pair with only BG-Lure^®^, and two pairs with the two attractants together (in diametrically opposite positions). The electric traps were exposed during the same collection period as the hand-net method, and the samples were stored after the end of both the morning and afternoon periods using the same procedure.

### 2.3. Identification of Material

In the laboratory, the biological material frozen in nitrogen was transferred to a freezer at −80 °C until the identification of the genus and species on a cold table at −20 °C. Taxonomic keys were used for the morphological identification of Culicidae [1,2,34,35].

### 2.4. Data Analysis

The data were entered into an information system operating on a web server developed especially for the project.

For the estimation of species diversity, species accumulation rate and statistical abundance analysis were performed using the EstimateS version 9 statistics program [36]. Diversity (S) was determined by the number of species collected per month in each environment and stratum and by the capture method. The accumulation curve was calculated using the Coleman rarefaction method. The estimate of the true number of species was performed with the Chao1 estimator and a confidence interval of 95%.

To compare diversity among the different methods (technique–attractant–stratum), the Shannon (H) and Gini–Simpson (1-D) indices were calculated; with Simpson’s dominance (D) calculated using EstimateS v.9. To compare abundance among the different methods (technique–attractant–stratum), the Wilcoxon test was applied for the difference between the medians at a significance level of 95%. For this purpose, SPSS v.25 software was used.

For the cluster analysis, we used dendrograms based on Bray–Curtis similarity, which considers the abundance of specimens. For the pairwise analysis of the association between methods, we used Spearman’s correlation with the PAST version 4.05 statistics program [37].

## 3. Results

We collected a total of 1555 Culicidae specimens in 20 taxa, of which 17 species belonged to 8 genera. Only 24 specimens were males, corresponding to the genus *Culex* and to the species *Aedes albopictus* and *Psorophora albigenu* (Table S1). Eighty-five percent of the specimens were captured from the ground.

*Aedes scapularis* was the most abundant species, followed by *Haemagogus janthinomys/capricornii*, *Psorophora albigenu*, *Sabethes albiprivus*, and *Ae. albopictus* (Table 1). All species, except *Limatus durhamii* and *Sabethes belisarioi*, were more abundant at the ground level (*n* = 1302) than in the canopy (*n* = 229), including *Hg. janthinomys/capricornii* and *Sa. albiprivus*, which were the dominant species in the canopy. *Haemagogus leucocelaenus* was a rare species at ground level, with only two specimens collected (Table 1). 

The hand-net capture technique obtained significantly higher yields than the automatic traps, both in the canopy and on the ground, accounting for 78.6% of the total of specimens collected in the canopy and 72.1% on the ground (Table 1 and Table S2). The traps using only CO_2_ and CO_2_ + BG-Lure^®^ obtained an intermediate yield and showed no significant difference between them in relation to the same stratum (Table S2). Traps using exclusively the BG-Lure^®^ attractant had the lowest yield in both strata (Table 1, Table S2 and Table S3). 

Regarding the indicators for richness, diversity, and dominance (Table 2), we observed a qualitative similarity between the canopy and ground strata, as the differences between each method in the canopy had the same pattern on the ground. In addition, the values of the same method in the canopy were close to those on the ground. The differences were small between the values for absolute richness and abundance for the different techniques and attractants in each stratum and, in general, with greater richness on the ground compared to the canopy. The biggest difference was in the trap results associated with BG-Lure^®^ in the canopy, where only one specimen was collected and, thus, presented very different indicators of the same method at ground level. Hand-net capture was the technique with greater richness and diversity and less dominance in both strata.

**Figure 2:**
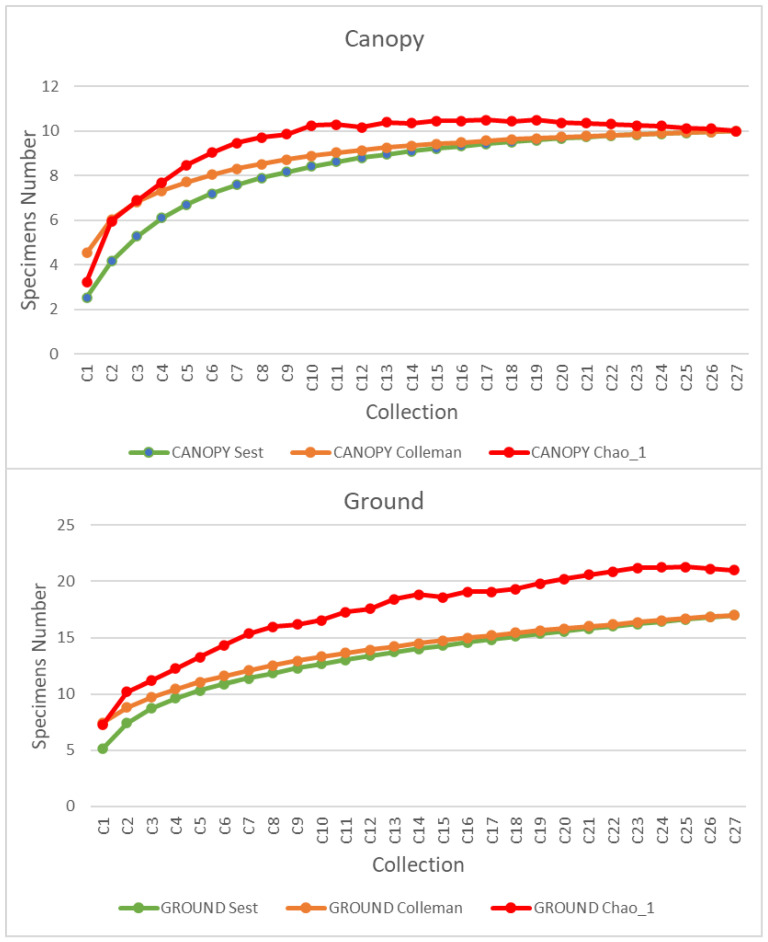
Species accumulation curves for Culicidae specimens collected in canopy and ground strata of the arborized savanna in the “Pé-do-Gigante” sector of the Vassununga State Park. Note: C = collection.

Species accumulation curves show a tendency to anticipate stability for the canopy compared to the ground (Figure 2). This stability in the canopy added to the qualitative and quantitative results, as seen in Table 1.

The analysis of the pair-to-pair comparison of different methods (stratum–technique–attractant) showed significance values between practically all methods, with the exception of the trap using only the BG-Lure^®^ installed in the canopy (with negative results) and the trap installed on the ground versus the traps with the CO_2_ + BG-Lure^®^ 1 and 2 in the canopy (Table 3). The highest coefficients occur between the traps using CO_2_ and CO_2_ + BG-Lure^®^ at the ground level and between the nets on the ground and in the canopy.

**Figure 3:**
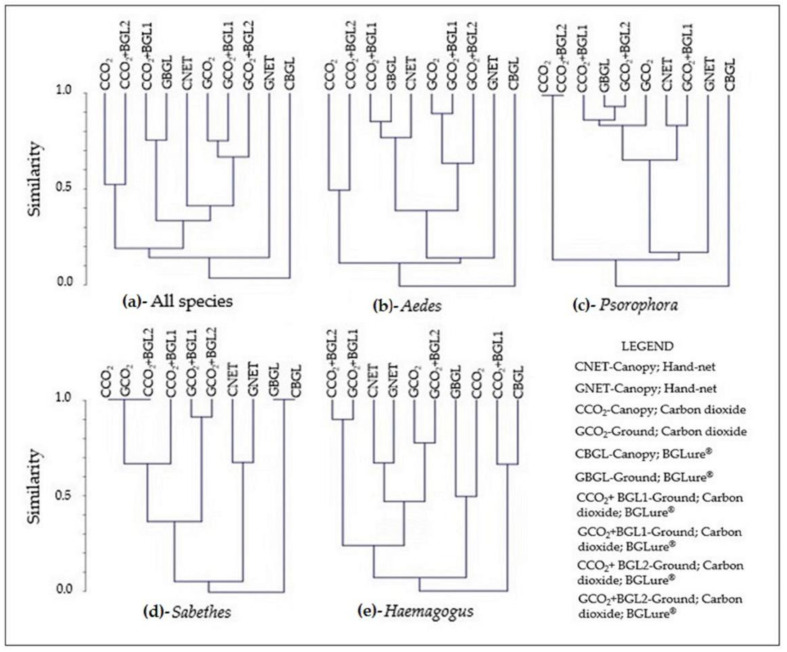
Bray–Curtis similarity dendrogram for different methods (technique–attractant–stratum) for the collection of Culicidae specimens in the “Pé-do-Gigante” sector of the Vassununga State Park, according to all identified species (**a**) and, according to the four genera with the highest number of specimens: (**b**)—*Aedes*; (**c**)—*Psorophora*; (**d**)—*Sabethes*; (**e**)—*Haemagogus*.

The Bray–Curtis similarity analysis for the different methods for collecting mosquito species is used to verify clusters of those methods that are most similar in qualitative and quantitative terms of species. Figure 3a shows the similarity dendrogram based on the analysis of all species found. A cluster that stands out includes all the traps at ground level that used CO_2_ (exclusively or with BG-Lure^®^) and the hand-net in the canopy, which suggests that one method could replace the other or serve as a replica. At the other extreme, the ground-trap methods and traps using only BG-Lure^®^ in the canopy have the fewest similarities, being isolated from the others. This, however, can be attributed to different reasons, the first being the method presented superior abundance and richness, and the latter being almost null.

The analysis considering each of the four most abundant genera in individualized dendrograms is presented in Figure 3b–e. The genus *Psorophora* presents a very similar large group comprising several methods; ground hand-net methods and traps with only BG-Lure^®^ in the canopy appear in isolation, following a pattern similar to the dendrogram for all species, which also presents the group of traps with CO_2_ in the canopy. The genus *Aedes* also shows some variation of this pattern for the central group, with fewer similarities than *Psorophora*. In the case of the *Haemagogus* and *Sabethes* genera, both presented a group with great similarity composed of nets in the canopy and on the ground, suggesting the human attractant as an aggregator. However, *Haemagogus* seems to be the genus that expresses more standardized responses to methods, while *Sabethes* shows more diffuse behavior for technique –attractant–stratum combinations.

## 4. Discussion

Our study is the most recent survey of diurnal mosquitoes in the savanna biome, where species richness and abundance were studied according to forest stratification. The main objective was to evaluate alternative capture methods to the hand-net, using the attractants BG-Lure^®^ and carbon dioxide in order to increase the effectiveness of automatic traps, especially for species of epidemiological interest that frequent the forest canopy. Thus, we sought to carry out collections in a favorable seasonal period, mainly for the species of the Aedini and Sabethini tribes [38,39,40,41,42,43].

Based on the results of the species accumulation curves for both strata, we verified that the sampling in the period was efficient, allowing for a good characterization of the diurnal mosquito fauna in the “Pé-do-Gigante” sector of the park. Thus, although we used a shorter collection period (27 days) than in other studies conducted in savanna areas (40–60 days) [3,44,45], we found similar species richness for the genera *Aedes*, *Haemagogus*, *Psorophora,* and *Sabethes*, with 14 species found in our study, and 14 to 17 in the others. It is also worth mentioning that these three studies were carried out in central Brazil, between latitudes 15.5 and 17.0°S, while our study, at latitude 21.5°S, represents an important record of this fauna in a region close to the southern limit of this biome in Brazil [46].

In addition, due to the methodology of simultaneous collection in the canopy and on the ground, a robust comparison between these two strata was possible. In other systematic studies [39,47] or specific entomological surveys associated with epidemic outbreaks [48,49,50] that also investigate the community of diurnal mosquitoes using the mobile human bait technique with hand-nets, collections were not carried out in the canopy. Thus, all the data on the abundance, richness, and dominance of species in the region were related to collections at the ground level. Our study provides pioneering results on the stratification of mosquito communities in the northwest region of the state of São Paulo.

Considering the general results from the two strata, we can observe the dominance of *Aedes scapularis* and the subdominance of *Haemagogus janthinomys/capricornii*, *Psorophora albigenu*, *Sabethes albiprivus,* and *Ae. albopictus*. In the comparison between strata, *Ae. scapularis* was dominant on the ground, while *Hg. janthinomys/capricornii* dominated in the canopy. Qualitatively, the list of species in the canopy and at ground level was very similar. In quantitative terms, practically all the species found in the canopy had a lower number of specimens compared to collections carried out on the ground, which was also observed in other studies carried out in the savanna [3,44,45].

This suggests that microclimatic conditions in the canopy may be less favorable than at ground level. We should emphasize that the collection points were in the “Cerrado *ss*” physiognomy, where the shading and size of the trees were less than in other physiognomies found in the park, such as the “Cerradão”, gallery forest, and semideciduous seasonal forest [31,51]. In this sense, the canopy was more subject to desiccation, affecting the intensity and maintenance of relative humidity, which is a key environmental factor for the main species found there [39,52,53].

The values of the indicators of diversity, uniformity, and dominance were similar for the same method between the canopy and the ground. The same was not observed for the richness and abundance indicators, which were higher in the soil. The Spearman’s correlation results corroborate this, since significant differences were found between the different methods in the same stratum and between strata. Contrarily, these indicators were different between the strata and methods in a similar study we carried out in the Atlantic Forest biome in Cantareira State Park [21].

However, in both studies, the hand-net was the technique with greater richness and diversity and lower dominance in both strata, while the indicators of traps using only BG-Lure^®^ demonstrated inefficiency, a result also found for wild diurnal mosquitoes in Brazil by other researchers [19,54]. This shows that the exclusive use of BG-Lure^®^ may be inefficient for wild neotropical species. The use of CO_2_ as an attractant demonstrated worse performance than its use together with BG-Lure^®^, a different result from that found in the Atlantic Forest [21] and probably due to the differences in specific environmental conditions in the savanna. 

It is worth mentioning that there are few attractants available for commercial use, as can be seen in many studies carried out in Brazil to evaluate the performance of mosquito collection with automatic traps [19,21,22,23,24,25,27,28,29,54,55]. This suggests the need to develop in situ studies using olfactometry to discover different substances that present selective attractiveness for each species of interest, according to the biome where it will be used. 

Among the most abundant species of mosquitoes collected in the “Pé-do-Gigante” sector, all of them showed epidemiological importance, especially for the transmission of yellow fever. *Haemagogus janthinomys*, *Hg. leucocelaenus*, and *Sa. chloropterus* are considered the main vector species in South America [56], the first two with the greatest range in Brazil [57,58]. In our study, we used the nomenclature *Haemagogus janthinomys/capricornii* because we were unable to determine which of the two species occurred at the site, since they are morphologically differentiated only by the male genitalia, and the investigated site is in the co-occurrence zone [2]. At any rate, whatever the species, it was the most abundant in the canopy compared to the other mosquito species, with a greater number of specimens at ground level than in the canopy. This result differs from several studies in which the species is cited as an acrodendrophile [19,45,52,59,60]. On the other hand, it adds to the results of other studies where variations in this pattern were observed [3,44,54].

The other species with epidemiological importance for yellow fever, which presented the greatest number of specimens, were *Ae. scapularis*, *Ae. albopictus*, *Ps. albigenu*, *Sa. albiprivus*, and *Sa. glaucodaemon*, and, to a lesser extent, *Ae. serratus*, *Ps. ferox*, and *Hg. leucocelaenus*. The rarity of *Hg. leucocelaenus* is noteworthy, since it is one of the dominant species in Cantareira State Park [21] and a vector for the transmission of the yellow fever virus in other areas of the Atlantic Forest [58,61,62]. Perhaps this species has no favorable habitat in the savanna, at least in the stricto sensu physiognomy investigated in this study. The assessment of its presence and abundance in enclaves of seasonal semideciduous forests, gallery forests, and in the physiognomy of the “Cerradão” deserves to be studied in order to better understand the details of the ecology of the species in this biome, since previous studies have not exactly focused on this [26,44,45,47,63].

An important result of this research is the assessment of the success of different techniques and attractants in the canopy and on the ground for capturing the genera *Aedes*, *Haemagogus*, *Psorophora*, and *Sabethes*, providing valuable data for arbovirus surveillance services in Brazil. In the similarity cluster analysis, the selectivity of hand-nets for the genera *Haemagogus* and *Sabethes* were evident in the dendrograms, while for the genera *Aedes* and *Psorophora*, the similarity between methods was more similar to the general dendrogram (Figure 3a all species), where the hand-net on the ground and the trap using only BG-Lure^®^ in the canopy appear distant from the other methods, with the former having the highest relative yield and the latter the lowest relative yield. 

In investigations of epidemics or epizootic outbreaks, the right place and time are crucial for obtaining good samples [10,64]. Thus, the use of the technique with the highest yield for the target species is very important. However, it is worth considering that the “ideal” collection technique is not always possible for surveillance services. Moreover, the use of automatic traps may be the only way to expand the space–time factor in routine and large-scale surveys [65,66]. Thus, if it is necessary to make use of these alternative techniques, this study, and that of Deus et al. (2022), may represent an important reference for the operational planning of field actions [21].

## 5. Conclusions

The results of this study highlight the benefits of using hand-nets over automatic traps with kairomones to capture diurnal mosquitoes, as they presented higher values of richness and abundance for species of epidemiological interest. Our research suggests that electric traps should be used to collect diurnal mosquitoes in the savanna when used with CO2 for species-richness studies of diurnal Culicidae. In the studied locality of “Cerrado ss”, collections at ground level showed greater abundance and species richness than the canopy. For surveys that require more abundance of specimens, it is necessary to consider the increase in effort in terms of the number of traps.

It is clear that there is still much to be explored concerning attractants, and it is important to encourage the development and testing of new kairomones associated with specific hosts, especially when the objective is to increase epidemiological and/or epizootic research based on the infectivity of mosquitoes and the genomic study of the etiologic agent. 

## Figures and Tables

**Table 1:** Number of Culicidae females collected in the “Pé-do-Gigante” sector of the Vassununga State Park, by forest stratum, technique, and attractant.

Taxa	Canopy							Ground							Total	
	BGL	CO_2_	CO_2_ + BGL1	CO_2_ + BGL2	NET	TOTAL	%	BGL	CO_2_	CO_2_ + BGL1	CO_2_ + BGL2	NET	TOTAL	%	N	%
*Aedes albopictus*					4	**4**	**1.7**	2	9	6	17	115	**149**	**11.4**	**153**	**10.0**
*Aedes scapularis*		3	12	1	18	**34**	**14.8**	14	65	78	34	408	**599**	**46.0**	**633**	**41.3**
*Aedes serratus*												2	**2**	**0.2**	**2**	**0.1**
*Coquillettidia juxtamansonia*												1	**1**	**0.1**	**1**	**0.1**
*Culex* sp.				1	7	**8**	**3.5**	1	1	4		22	**28**	**2.2**	**36**	**2.4**
*Haemagogus janthinomys/capricornii*		1	2	1	69	**73**	**31.9**		1	6	5	134	**146**	**11.2**	**219**	**14.3**
*Haemagogus leucocelaenus*												2	**2**	**0.2**	**2**	**0.1**
*Limatus durhamii*					1	**1**	**0.4**								**1**	**0.1**
*Psorophora albigenu*		1	11	1	16	**29**	**12.7**	8	10	18	9	123	**168**	**12.9**	**197**	**12.9**
*Psorophora discrucians*									1				**1**	**0.1**	**1**	**0.1**
*Psorophora ferox*									1			3	**4**	**0.3**	**4**	**0.3**
*Psorophora varipes*										4		3	**7**	**0.5**	**7**	**0.5**
*Psorophora* sp.								1				3	**4**	**0.3**	**4**	**0.3**
*Sabethes albiprivus*		3		5	35	**43**	**18.8**	1	26	7	18	92	**144**	**11.1**	**187**	**12.2**
*Sabethes belisarioi*				1	5	**6**	**2.6**			1		3	**4**	**0.3**	**10**	**0.7**
*Sabethes glaucodaemon*	1		2	3	22	**28**	**12.2**		2	3	5	22	**32**	**2.5**	**60**	**3.9**
*Sabethes purpureus*					3	**3**	**1.3**		2		1	4	**7**	**0.5**	**10**	**0.7**
*Sabethes tarsopus*												1	**1**	**0.1**	**1**	**0.1**
*Sabethes* sp.									1	1			**2**	**0.2**	**2**	**0.1**
*Wyeomyia confusa*												1	**1**	**0.1**	**1**	**0.1**
**Total**	**1**	**8**	**27**	**13**	**180**	**229**	**100.0**	**27**	**119**	**128**	**89**	**939**	**1302**	**100.0**	**1531**	**100.0**
**%**	**0.4**	**3.5**	**11.8**	**5.7**	**78.6**	**100.0**		**2.1**	**9.1**	**9.8**	**6.8**	**72.1**	**100.0**		**100.0**	
	**15.0**							**85.0**								

Techniques/attractants: BGL, CDC with only BG-Lure^®^; CO_2_, CDC with only CO_2_; CO_2_ + BGLl or CO_2_ + BGL2, CDC with CO_2_ + BG-Lure^®^; NET, hand-net.

**Table 2:** Indicators of abundance, richness, diversity, and dominance of Culicidae specimens collected in the “Pé-do-Gigante” sector of the Vassununga State Park, by forest strata, technique, and attractant. Next page.

Parameter	Canopy					Ground				
	BGL	CO_2_	CO_2_ + BGL1	CO_2_ + BGL2	NET	BGL	CO_2_	CO_2_ + BGL1	CO_2_ + BGL2	NET
S (Taxa)	1	4	4	7	10	5	10	9	7	16
N (Abundance)	1	8	27	13	180	26	118	127	89	936
D (Dominance)	1	0.31	0.37	0.23	0.22	0.39	0.37	0.41	0.24	0.25
1-D (Geni-Simpson Index)	0	0.69	0.63	0.77	0.78	0.61	0.63	0.59	0.76	0.75
H (Shannon Index)	0	1.26	1.11	1.69	1.80	1.14	1.37	1.37	1.61	1.70

Techniques/attractants: BGL, CDC with only BG-Lure^®^; CO_2_, CDC with only CO_2_; CO_2_ + BGLl or CO_2_ + BGL2, CDC with CO_2_ + BG-Lure^®^; NET, hand-net.

**Table 3:** Spearman’s correlation coefficients (**ρ**) and statistical significance (*p*) values from the pair-to-pair comparison between different methods (stratum–technique–attractant) for mosquito collection in the “Pé-do-Gigante” sector of the Vassununga State Park.

Strata		Canopy					Ground				
	Methods	BGL	CO_2_	CO_2_ + BGL1	CO_2_ + BGL2	NET	BGL	CO_2_	CO_2_ + BGL1	CO_2_ + BGL2	NET
			***p* valor**								
**Canopy**	**BGL**		0.622	0.096	0.101	0.184	0.571	0.520	0.853	0.492	0.536
	**CO_2_**	−0.12		**0.001**	**0.011**	**0.001**	**0.004**	**0.004**	**0.001**	**0.001**	**0.001**
	**CO_2_ + BG1**	0.39	0.68		**0.024**	**0.002**	0.064	**0.022**	**0.008**	**0.008**	**0.002**
	**CO_2_ + BG2**	0.39	0.57	0.52		**0.000**	0.096	**0.006**	**0.000**	**0.004**	**0.040**
	**NET**	0.32	0.69	0.66	**0.73**		**0.011**	**0.005**	**0.003**	**0.002**	**0.000**
**Ground**	**BGL**	−0.14	0.63	0.43	0.39	0.57		**0.001**	**0.001**	**0.002**	**0.001**
	**CO2**	0.16	0.62	0.52	0.60	0.62	**0.71**		**0.000**	**0.000**	**0.004**
	**CO_2_ + BG1**	0.05	0.69	0.59	**0.75**	0.65	**0.71**	**0.74**		**0.000**	**0.002**
	**CO_2_ + BG2**	0.17	**0.70**	0.59	0.63	0.67	0.66	**0.91**	**0.81**		**0.002**
	**NET**	0.15	0.68	0.66	0.47	**0.81**	0.69	0.63	0.67	0.65	
		**ρ valor**									

Techniques/attractants: BGL, CDC with only BG-Lure^®^; CO_2_, CDC with only CO_2_; CO_2_ + BGLl or CO_2_ + BGL2, CDC with CO_2_ + BG-Lure^®^; NET, hand-net.

## Data Availability

Not applicable.

## Supplementary Materials

The following supporting information can be downloaded at: https://www.mdpi.com/article/10.3390/tropicalmed7120446/s1, Table S1: Number of male specimens collected by genus and species, according to stratum (canopy and ground) and capture technique (treatment). Santa Rita do Passa Quatro, SP, Brazil.; Table S2: Results of the pairwise comparison between methods and strata by the Wilcoxon test; Table S3: Descriptive analysis results; File S1: Photos of Platforms and CDCs Installed; File S2: Description Collection Equipment: Hand-Net and Mouth Aspirator.
