# Supplementary material for: Evaluation of Methods for Collecting Mosquitoes (Culicidae: Diptera) in Canopy and Ground Strata in the Brazilian Savanna
Source: Trop Med Infect Dis. 2022 Dec 19;7(12):446. doi: 10.3390/tropicalmed7120446 (PMC9783515; doi:10.3390/tropicalmed7120446)

Tropical Medicine and Infectious Disease

MDPI

Article

Supplementary Materials for Evaluation of Methods for Collecting Mosquitoes (Culicidae: Diptera) in Canopy and Ground Strata in the Brazilian Savanna

Table S1. Number of male specimens collected by genus and species, according to stratum (canopy and ground) and capture technique (treatment). Santa Rita do Passa Quatro, SP, Brazil.

Specie	N	Method	Stratum
Culex sp	8	net	ground /canopy
Aedes albopictus	13	net	ground
Psorophora albigenu	3	net/BGL*	ground
**Total**	24		

* net, hand-net; BGL, CDC with only BG-Lure®.

Table S2. Results of the pairwise comparison between methods and strata by the Wilcoxon test.

Strata	Methods	Canopy					Ground				
		BGL	CO2	CO2+ BGL1	CO2+ BGL2	NET	BGL	CO2	CO2+ BGL1	CO2+ BGL2	NET
Canopy	BGL		0.072	**0.011**	**0.027**	**0.000**	**0.020**	**0.000**	**0.001**	**0.000**	**0.002**
	CO2			0.858	0.526	**0.002**	0.763	**0.001**	**0.001**	**0.007**	**0.000**
	CO2+BGL1				0.525	**0.001**	0.856	**0.005**	**0.004**	**0.014**	**0.000**
	CO2+BGL2					**0.002**	0.241	**0.002**	**0.014**	**0.006**	**0.000**
	NET						**0.002**	0.489	**0.000**	0.222	**0.000**
Ground	BGL							**0.005**	**0.009**	**0.006**	**0.000**
	CO2								0.477	0.431	**0.000**
	CO2+BGL1									0.394	**0.000**
	CO2+BGL2										**0.000**
	NET										

Techniques/attractants: BGL, CDC with only BG-Lure®; CO_2, CDC with only CO_2; CO_2 + BGLl or CO_2 + BGL2, CDC with CO_2 + BG-Lure®; NET, hand-net.

Table S3. Descriptive analysis results.

Strata	Method	Collection	Minimum	Maximum	Average	Error Average	Error Deviation	Variance
Canopy	BGL	27	0	1	0.04	0.037	0.192	0.037
	CO2	27	0	29	1.37	1.076	5.589	31.242
	CO2+BGL1	27	0	18	1.00	0.671	3.486	12.154
	CO2+BGL2	27	0	7	0.81	0.311	1.618	2.618
	NET	27	0	28	6.67	1.639	8.517	72.538
Ground	BGL	27	0	22	1.00	0.811	4.215	17.769
	CO2	27	0	31	6.07	1.801	9.356	87.533
	CO2+BGL1	27	0	44	7.33	2.093	10.877	118.308
	CO2+BGL2	27	0	23	4.48	1.377	7.154	51.182
	NET	27	0	115	35.04	6.429	33.404	1115.806

File S1: Photos of Platforms and CDCs Installed

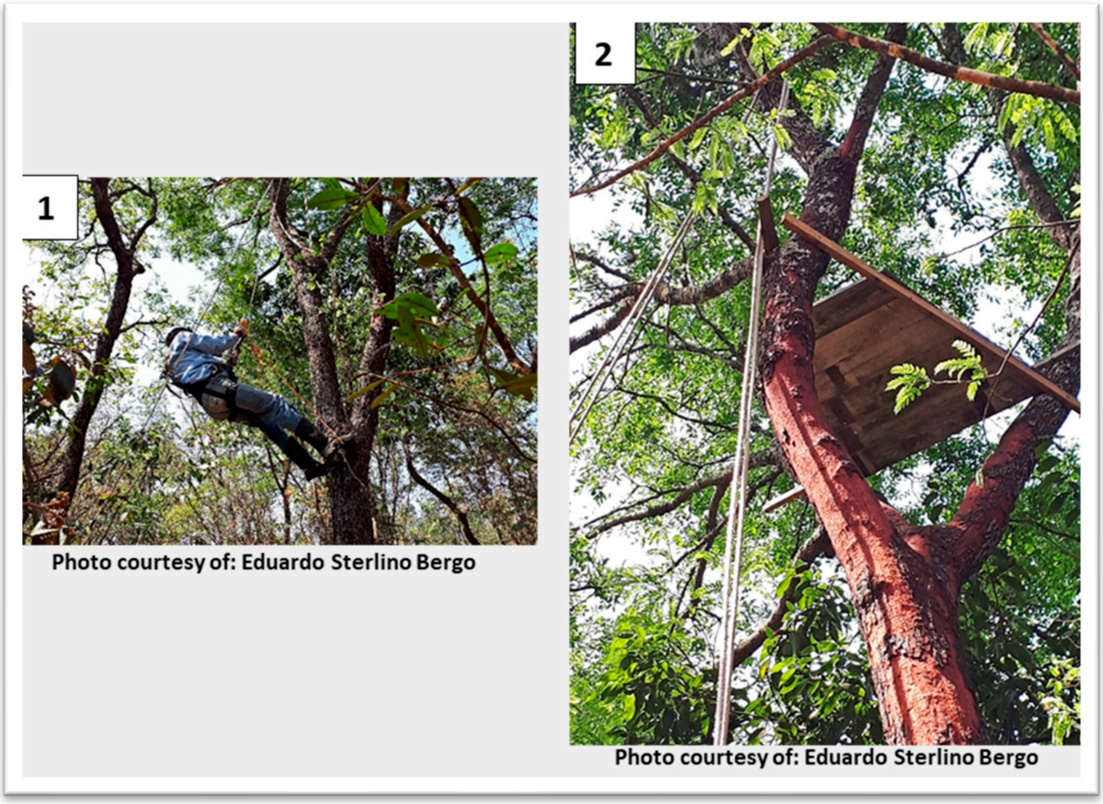

Photos 1 and 2—Preparing and installing the platform.

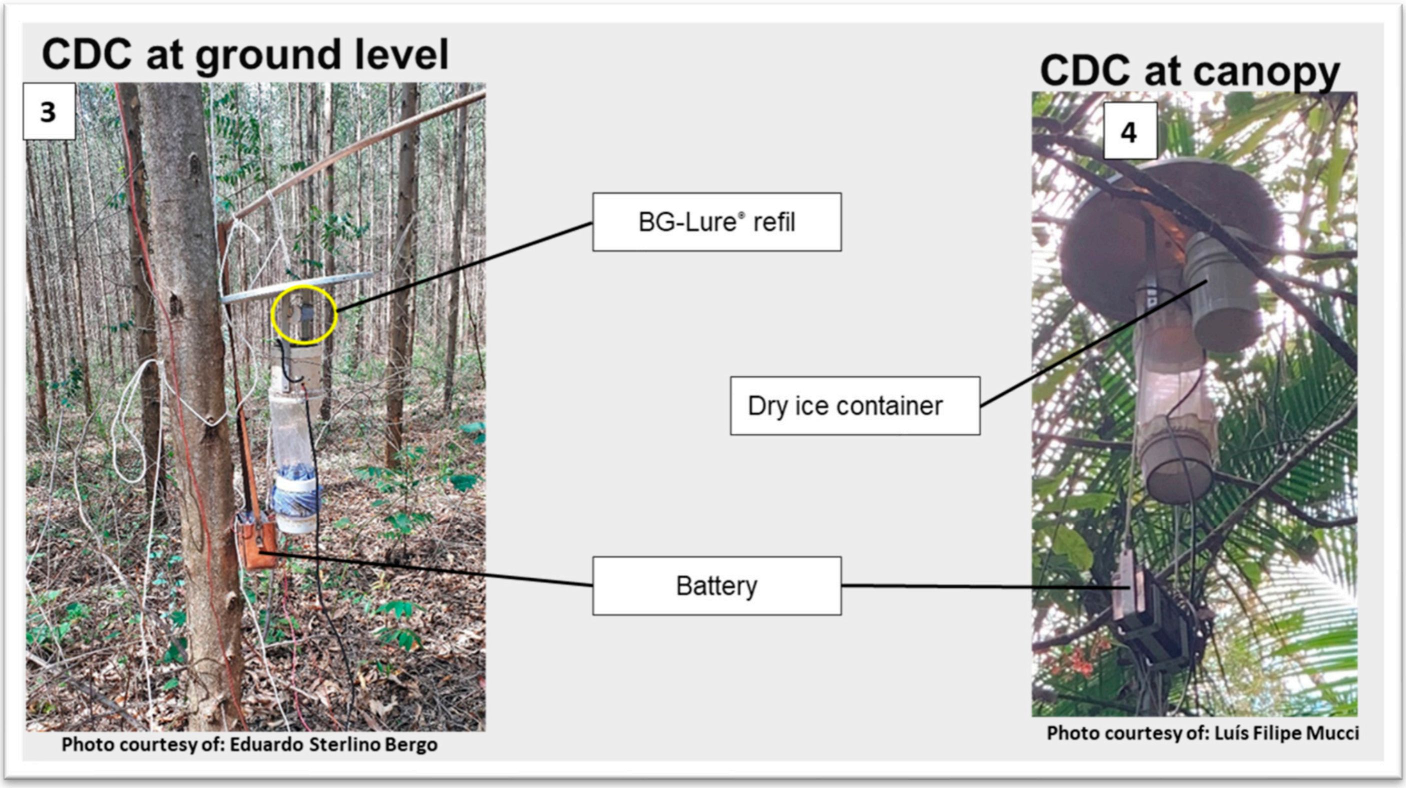

Photos 3 and 4—CDC trap, highlighting the batteries and the attractant.

File S2. Description Collection Equipment: Hand-Net and Mouth Aspirator

Adapted from Brasil, M. da S. *Guia de Vigilância de Epizootias Em Primatas Não Humanos e Entomologia Aplicada à Vigilância Da Febre Amarela*; 2nd ed.; Ministério da Saúde, Secretaria de Vigilância em Saúde, Departamento de Vigilância das Doenças Transmissíveis, 2014. ISBN 978-85-334-2102-8. Available in: https://bvsms.saude.gov.br/bvs/publicacoes/guia_vigilancia_epizootias_primatas_entomologia.pdf. [Accessed on: July 1st, 2022].

1) Hand-net for mosquitoes capture: fabricated with a rod made of resistant aluminum. It is made of voile (polyester) with a conical shape and tightly closed and resistant weaves, with a seam on the wrong side along the length of the basket and with a very tight seam along the diameter of the rod; the end of the basket is straight and 4cm wide.

Dimensions:

- Cable length of PVC (polyvinyl chloride)-coated cable: 15 cm
- Basket length: 39 cm
- Basket inner diameter: 19 cm

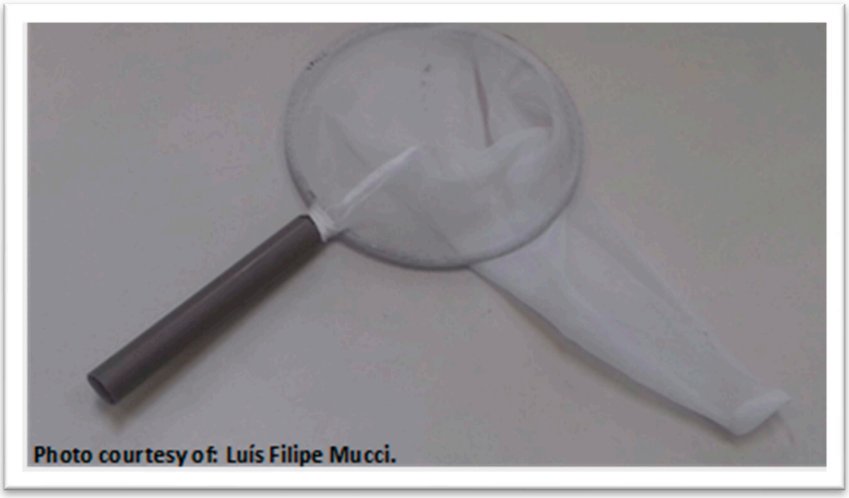

2. Aspirator for winged mosquitoes with suction base storage, comprising:

• 15 cm test tube (glass or PVC—translucent);

• Attached to the tube is a rubber stopper, approximately 2.6 cm high, 2.4 cm in diameter, and 2 cm in diameter, containing two holes with: a hole to attach a latex tourniquet that is 1 m long with an internal diameter of approximately 5 mm, while the ends of the tourniquet contain one rigid transparent plastic tube of 8 cm for suction; at the other end, which is inside the test tube, there is another rigid plastic with a filter voile on the tip. In the other, there is a hole with a 30 cm rigid-plastic tube whose external end contains a 5x3 cm plastic horn whistle.

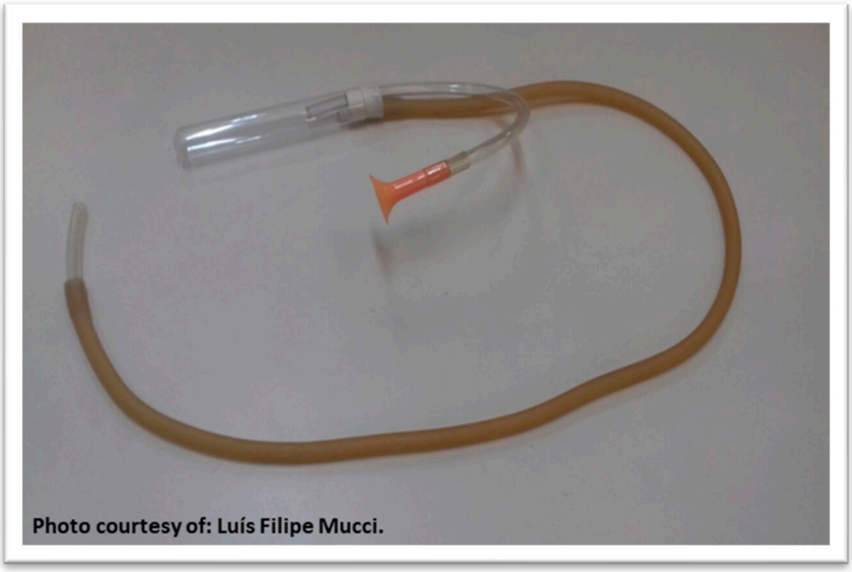

Supplement: Supplementary file 1 [file tropicalmed-07-00446-s001.zip › tropicalmed-1989591-supplementary.pdf]
